# The interaction of supramolecular anticancer drug amphiphiles with phospholipid membranes†

**DOI:** 10.1039/d0na00697a

**Published:** 2020-10-26

**Authors:** Phu K. Tang, Anjela Manandhar, William Hu, Myungshim Kang, Sharon M. Loverde

**Affiliations:** Department of Chemistry, College of Staten Island, City University of New York 2800 Victory Blvd., 6S-238 Staten Island NY 10314 USA sharon.loverde@csi.cuny.edu; Ph.D. Program in Biochemistry, The Graduate Center of the City University of New York New York USA; Hunter College High School New York NY 10128 USA; Ph.D. Program in Chemistry and Physics, The Graduate Center of the City University of New York New York USA

## Abstract

The shape of drug delivery vehicles impacts both the circulation time and the effectiveness of the vehicle. Peptide-based drug amphiphiles (DAs) are promising new candidates as drug delivery vehicles that can self-assemble into shapes such as nanofilament and nanotube (diameter ∼ 6–10 nm). The number of conjugated drugs affects the IC50 of these DAs, which is correlated to the effective cellular uptake. Characterizing and optimizing the interaction of these DAs and their assemblies with the cellular membrane is experimentally challenging. Long-time molecular dynamics simulations can determine if the DA molecular structure affects the translocation across and interaction with the cellular membrane. Here, we report long-time atomistic simulation on Anton 2 (up to 25 μs) of these DAs with model cellular membranes. Results indicate that the interaction of these DAs with model cellular membranes is dependent on the number of conjugated drugs. We find that, with increased drug loading, the hydrophobic drug (camptothecin) builds up in the outer hydrophobic core of the membrane, pulling in positively charged peptide groups. Next, we computationally probe the interaction of differing shapes of these model drug delivery vehicles—nanofilament and nanotube—with the same model membranes, finding that the interaction of these nanostructures with the membrane is strongly repulsive. Results suggest that the hydrogen bond density between the nanostructure and the membrane may play a key role in modulating the interaction between the nanostructure and the membrane. Taken together, these results offer important insights for the rational design of peptide-based drug delivery vehicles.

## Abstract 2

Molecular dynamics simulations probe drug delivery vehicle-membrane interaction.

## Graphical abstract

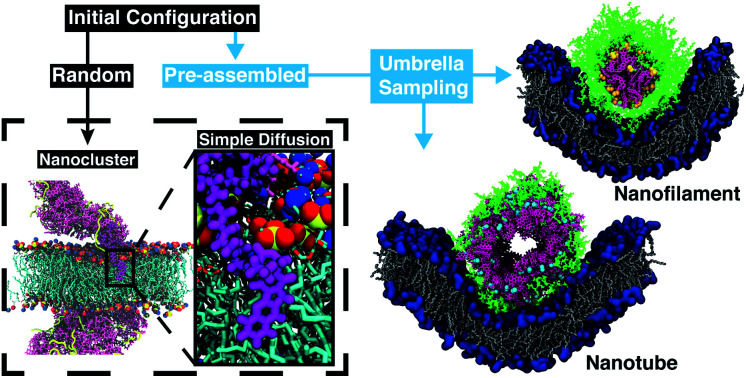

## Introduction

There are many challenges in the design of an effective drug delivery vehicle, including a controlled and high drug loading capacity, extending the circulation time in blood-stream,^1^ eliminating non-specific cell uptake,^2^ tunability of the vehicle morphology/shape at the nanoscale,^3^ and ultimately control of the vehicle interaction with the cellular membrane—either through active targeting of cellular receptors and/or control of the membrane response *via* morphology^4^ and/or surface patterning.^5,6^ Small molecules such as ions, peptides, and sugars (<1 kDa), dependent on their size and polarity, can cross the membrane *via* a passive diffusion or else an active transport mechanism.^7^ In contrast, large molecules such as proteins and viruses (≥10 kDa) traverse the membrane barrier either through pore formation or endocytosis.^8^ The relative contributions to the free energy of interaction between the particle (large molecule such as protein or virus) and the membrane include the bending energy of the membrane, the membrane tension, as well as any adhesive contact between the particle and membrane.^9^ Particle-based simulation methods such as molecular dynamics offer an emerging tool to probe nanoparticle–membrane interactions,^10,11^ characterizing, for example, the effect of size,^12^ shape,^13^ surface charge,^14^ and chemistry of the nanoparticle.^15^ For example, molecular simulations can uniquely capture deformations and rearrangements of the nanoparticle itself at the molecular level, such as molecular ‘snorkeling’,^15^ which cannot be well-described with continuum models. Ultimately, design of an effective drug delivery vehicle entails engineering not only the shape of the vehicle, but the interaction with the cellular membrane.

**Fig. 1:**
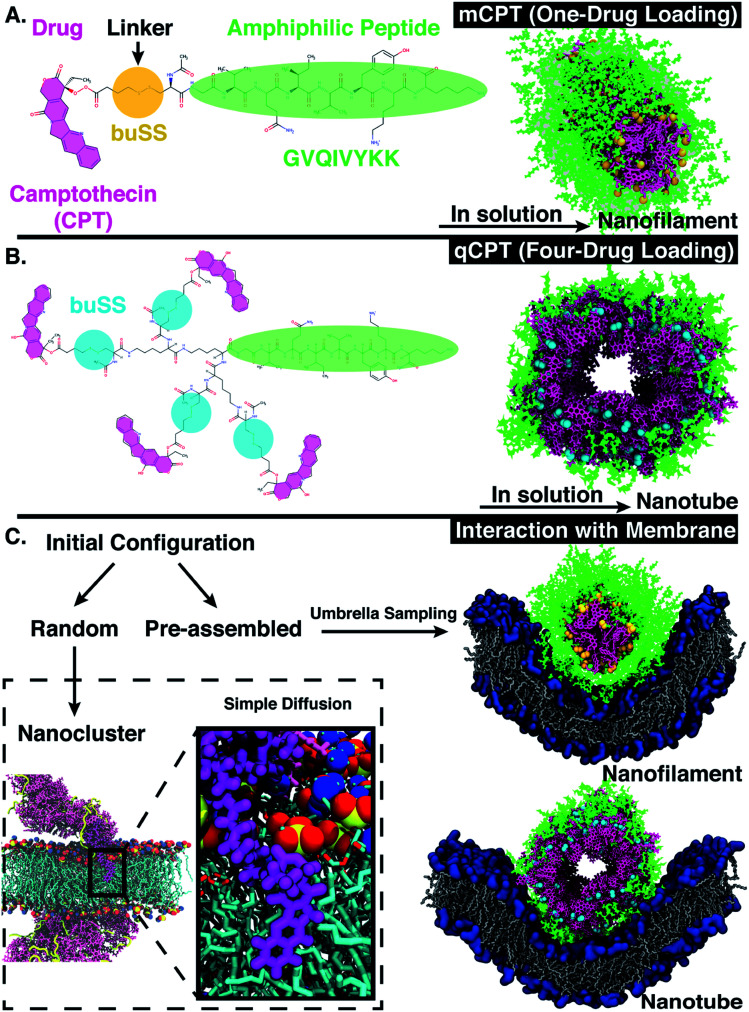
(A) Chemical structure of ‘mCPT-buSS-Tau’, which has one-drug loaded per peptide. In solution, ‘mCPT-buSS-Tau’ can self-assemble into a nanofilament. Camptothecin (CPT), linker, and peptide are highlighted in magenta, yellow, and green, respectively. (B) Chemical structure of ‘qCPT-buSS-Tau’. In solution ‘qCPT-buSS-Tau’ can self-assemble into a nanotube. CPT, linker, and peptide are highlighted in magenta, blue, and green, respectively. (C) Random initial configurations of ‘mCPT-buSS-Tau’ and ‘qCPT-buSS-Tau’ interact with a model membrane using unbiased molecular dynamics simulations (or simple diffusion). CPT (in magenta) and peptide (in yellow) are shown in ‘Licorice’ and ‘New Cartoon’ representation, respectively. The zoomed-in view shows a insertion of CPT into the model POPC (1-palmitoyl-2-oleoyl-*sn-glycero*-3-phosphocholine) membrane (head groups: van der Waals representation, colors: choline (blue) and phosphate (red, yellow)). Pre-assembled initial configurations of the nanofilament and the nanotube interact with the membrane using umbrella sampling. The head groups of the membrane are represented in dark blue ‘QuickSurf’ representation and the grey acyl tails in ‘Licorice’ representation.

Peptide amphiphiles (PAs) are a class of peptide-based molecules composed of hydrophilic head and hydrophobic tail^16–18^ that self-assemble into ordered nanostructures of various morphologies, such as ribbons, bilayers, tubes, and fibers.^19–22^ The morphology of the self-assembled nanostructure is driven at the molecular level by the balance of hydrophobicity and hydrophilicity, which can be further tuned by the peptide sequence. Peptide sequence can be tailored for various biomedical purposes such as stabilizing membrane proteins,^23,24^ facilitating cell differentiation,^25^ as well as serving as drug delivery vehicles.^26,27^ Indeed, peptides, such as ‘cell-penetrating peptides’ or CPPs, are classic examples of molecules that can be tailored *via* sequence to affect their membrane interaction. For example, certain functional domains^28^ control the translocation activity.^29^ As a result, these findings also act as general guidelines for rational designs of CPPs as potent drug delivery tools.^30–32^ Additionally, CPPs, such as a non β-sheet forming peptide from the protein transduction domain of Tat, HIV protein,^33^ can also be coupled to hydrophobic drugs to improve their delivery effectiveness.^34^ For example, Cui *et al.*^35^ designed peptide-based drug amphiphiles (DAs), consisting of a short modified Tau peptide sequence (CGVQIVYKK)^36^—the β-sheet forming segment of Tau (protein that stabilizes microtubules^37^)—and hydrophobic anticancer drug camptothecin (CPT), conjugated *via* a biodegradable disulfide linker (buSS)^38,39^ as shown in Fig. 1A and B. The sequence, CGVQIVYKK, is derived from a model peptide sequence (VQIVYK) that forms both parallel and anti-parallel β-sheets as characterized by Kirschner *et al.*^36^ Various peptide sequences have been used in constructing peptide amphiphiles as reviewed in Manandhar *et al.*^40^ Indeed, drug conjugates that employ disulfide bonds can be reduced at varying locations within cells.^38^ Nonetheless, conventional MD simulations cannot take into account the reversible dynamics of the disulfide bonds. CPT is a model hydrophobic anticancer drug^41^ with a known octanol/water partition coefficient^42,43^ that can π–π stack in solution due to its planar aromatic structure,^44^ driving the self-assembly process. Dependent on the molecular structure of the DAs, such as the number of conjugated drugs (‘mCPT’ or ‘mCPT-buSS-Tau’, which has one drug attached, *vs.* ‘qCPT’ or ‘qCPT-buSS-Tau’, which has four drugs attached), the shape of the self-assembly in solution ranges from nanofilament (diameter ∼ 6.5 nm) to nanotube (diameter ∼ 9.5 nm).^35^ Essentially, varying the drug loading varies the relative balance of hydrophobic to hydrophilic interactions, which determines the relative shape and diameter of these unidimensional self-assemblies.

In this article, we computationally investigate the dynamic self-assembly and stability of peptide-drug amphiphiles (DAs), as well as characterize their interactions with a model cellular membrane. We find that with increased drug loading singular DAs penetrate the membrane at short time scales (0.5 μs) due to their increased hydrophobicity and increased accessibility of the drug (Fig. 1C). Using advanced sampling methods in molecular dynamics, we find that self-assembled DA nanostructures—nanofilament and nanotube—repel the model membrane, forcing the membrane to thin and bend. This computational approach can be extended to molecularly design further self-assembled drug carriers, as well as predict the method of drug transport across the cellular membrane. With additional molecular design, control of the self-assembly shape and interaction with and transport across the cellular membrane of these DA's can then be optimized.

## Computational methods

### System setups

The phospholipid bilayers were preassembled and minimized using CHARMM-GUI.^45^ The 1-palmitoyl-2-oleoyl-*sn-glycero*-3-phosphocholines or POPC phospholipids were parameterized using the Amber Lipid14 (ref. 46) force field. Next, the bilayer was further equilibrated, in NAMD 2.13,^47^ with an anisotropic Langevin barostat^48,49^ using an oscillation period and decay time of 100 fs and 50 fs, respectively, for ∼100 ns. A damping coefficient of *γ* = 1 ps^−1^, at a pressure of 1 atm, was used together with the Langevin barostat to allow fluctuations of the membrane. ‘mCPT-buSS-Taau’ and ‘qCPT-buSS-Tau’ were parameterized with the General Amber Force Field (GAFF).^50^ TIP3P water was used. Packmol^51^ was used to set-up the initial configuration where the DAs were placed randomly above and below the POPC bilayer membrane. The ‘mCPT-buSS-Tau’ and ‘qCPT-buSS-Tau’ with POPC membrane systems were equilibrated for 80 ns and 88 ns respectively. The nanofilament/nanotube systems were constructed by combining a pre-equilibrated POPC membrane and the ‘mCPT-buSS-Tau’ nanofilament or the ‘qCPT-buSS-Tau’ nanotube. The nanofilament was constructed in the same way as previous nanofilaments reported by Kang *et al.*,^52^ specifically the nanotube where all four CPT drugs are parallel to each other. All systems used *tleap* from AmberTools^53^ to neutralize the overall charge with Cl^−^ ions. All system details/sizes are listed in Table 1.

**Table 1:** System details/sizes

Initial Configuration	Random		Pre-assembled	
System	mCPT	qCPT	Nanofilament	Nanotube
Box size, Å^3^	127 × 131 × 177	128 × 126 ×143	160 × 158 × 207	123 × 196 × 210
Molecules of POPC	500	500	505	505
Molecules of water	72 929	46 000	90 916	87 089
Molecules of Cl^−^	54	108	336	144
Molecules of DA	54	54	168	72
Bulk [DA], mM	39.3	54.7	N/A	N/A

### MD simulation parameters

All systems used the NPT ensemble with Langevin dynamics^48,49^ with a temperature of 310 K. A damping coefficient of *γ* = 1 ps^−1^, at a pressure of 1 atm, was used together with an anisotropic barostat to allow fluctuations of the membrane with a piston period of 100 fs and a damping time scale of 50 fs. The SHAKE algorithm^54^ was used to fix hydrogen atoms allowing a 2 fs timestep. The Particle Mesh Ewald (PME) algorithm^55^ was utilized to take full electrostatic interactions into account, with full periodic boundary conditions. The cut-off for van der Waals interactions was 12 Å with a smooth switching function at 10 Å. Bonded atoms were excluded from non-bonded atom interactions using a scaled 1–4 value. After equilibration, all random systems were transferred to Anton 2 ^56^ for production runs. During production runs on Anton 2, the timestep was changed to 2.5 fs. The short-range electrostatics was calculated every timestep while the long-range electrostatics was calculated every three timesteps. The Gaussian Split Ewald method^57^ was used to accelerate the electrostatic calculations. All other parameters were used as suggested by the Anton2 website (wiki.psc.edu/twiki/viewauth/Anton/WebHome). A production run of 18 μs was performed for the ‘mCPT-buSS-Tau’-POPC system and 25 μs for the ‘qCPT-buSS-Tau’-POPC system.

### Umbrella sampling

Here, we use Umbrella Sampling (US)^58^ to determine the interaction free energy of the ‘mCPT-buSS-Tau’ nanofilament and the ‘qCPT-buSS-Tau’ nanotube with the POPC membrane. US is one of the widely used method to calculate free energy for various biophysical properties such as – protein folding,^59,60^ peptide–peptide interactions,^61,62^ peptide–DNA interactions,^63^ and peptide–membrane interactions.^64^ The reaction coordinate (*r*_c_) is the *z*-distance between the center of mass of the nanofilament/nanotube and the membrane which is divided into multiple windows. For each US window a biasing harmonic potential (*w*_*i*_) is applied such that the states samples near the center of the window. The *w*_*i*_ in each window is defined as1
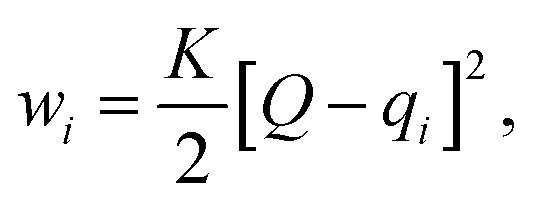
where *k* is the spring constant, *q*_*i*_ is the center and *Q* is the actual difference of *r*_c_ for initial and final state. The biased distribution function for every *i*^th^ window is defined as

2 〈*p*(*ξ*)〉^biased^_*i*_ = e−^*w*_*i*_(^ξ^)/*k*_B_*T*^〈*p*(ξ)〉〈e−^*w*_*i*_(^ξ^)/*k*_B_*T*^〉^−1^,

and the unbiased PMF is defined as

3 *W*_*i*_(*ξ*) = −*k*_B_*T* × ln 〈*p*(*ξ*)〉 − *w*_*i*_ − *F*_*i*_,

where *F*_*i*_ is the unknown free energy constant, defined as

4 e^*F*_i_/*k*_B_*T*^ = 〈e^*w*_i_/*k*_B_*T*^〉,

Next, the weighted histogram analysis method (WHAM)^65^ is used to determine *F*_*i*_. This uses an iterative process by making initial guess of *F*_*i*_ to estimate unbiased probability distribution as5
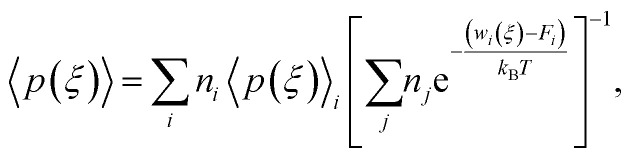


The resulting probability is then used to get new set of *F*_*i*_ values,6
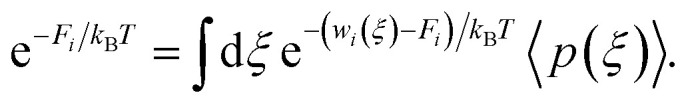


Here, we performed steered MD^66^ (SMD) simulations to pull the nanofilament and the nanotube with 7 kcal mol^−1^ force towards the POPC membrane to generate the initial positions in each window. Thus, the nanofilament interaction with the membrane has 38 windows 1 Å apart with *r*_c_ varying 70.5 Å to 33.5 Å (Fig. 7). Similarly, the nanotube interaction with the membrane has 49 windows 1 Å apart with *r*_c_ varying 84.5 Å to 36.5 Å (Fig. 8). Each configuration is harmonically constrained with spring constant of 20 kcal mol^−1^. These simulations have periodic boundary conditions, thus infinitely long nanofilament/nanotube is interacting with an infinitely wide cell membrane. For the nanostructure-membrane systems, same MD parameters as that for random drug amphiphile-membrane systems are used.

## Results

### Low drug loading and interaction with a POPC membrane

**Fig. 2:**
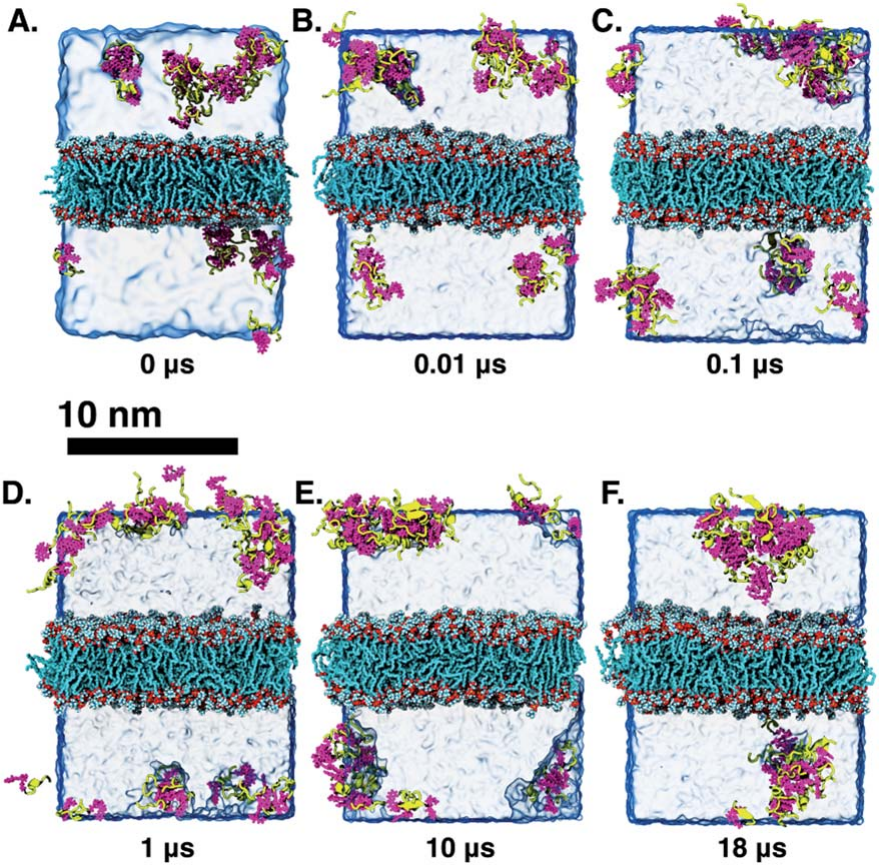
(A–F) Snapshots along the 18 microsecond trajectory showing nanoclusters of ‘mCPT-buSS-Tau’ interacting with the model POPC (1-palmitoyl-2-oleoyl-*sn-glycero*-3-phosphocholine) membrane. CPT (Camptothecin) are in magenta with ‘Licorice’ representation, peptides are yellow with a ‘Secondary Structure’ representation. The linkers are in black. POPCs is shown in cyan, red, and white in a ‘Licorice’ representation. The water is shown with a transparent box.

**Fig. 3:**
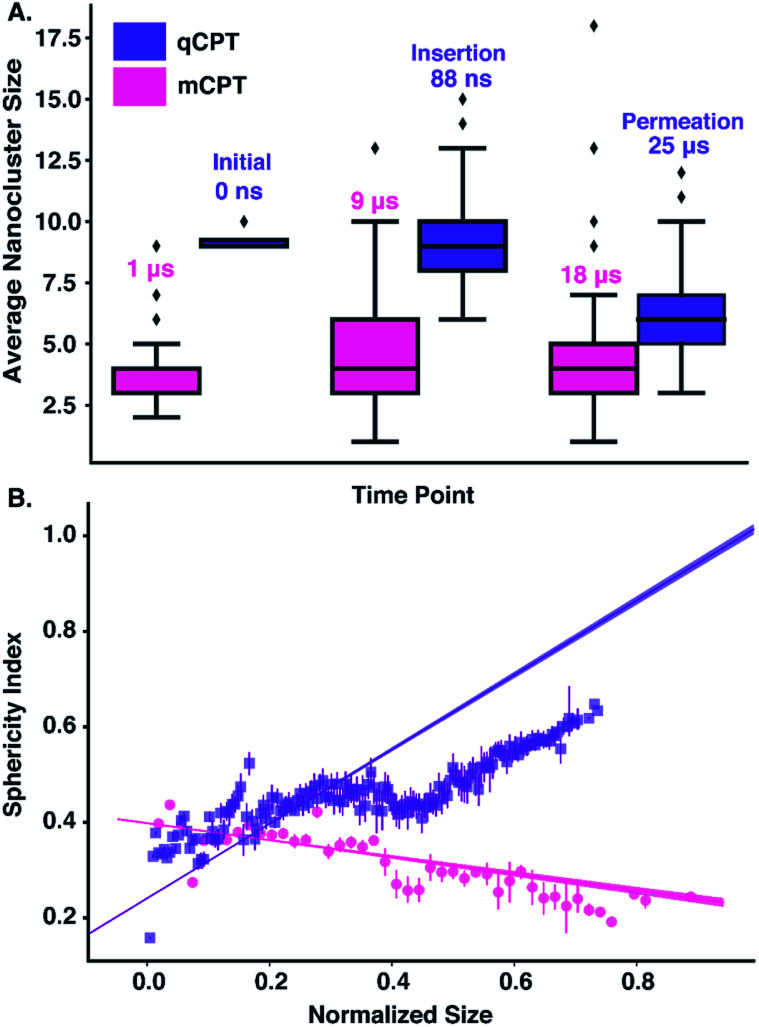
(A) The average size of ‘mCPT-buSS-Tau’ and ‘qCPT-buSS-Tau’ nanoclusters based on drug aggregation is shown after three time points for ‘mCPT-buSS-Tau’ (magenta) (1 μs, 9 μs, and 18 μs) and ‘qCPT-buSS-Tau’ (purple): ‘Initial’ (0 μs), ‘Insertion’ (88 ns), and ‘Permeation’ (18 μs). The lowest, middle, and top lines of the boxes are 25, 50, and 75 percentiles, respectively. The top and bottom lines are maxima and minima, with the outliers as black diamonds. (B) The sphericity index as a function of normalized size. The error bars are shown as straight lines. Linear regressions with 95% confident interval are shown.

Long-time molecular dynamics can assess if the DA structure affects the translocation across and interaction with the cellular membrane. To begin with, studies on *Anton2* ^56^ indicate that the interaction of ‘mCPT-buSS-Tau’ with model cellular membranes is repulsive, and that small aggregates of the DAs do not interact with or disturb the membrane as shown in Fig. 2, even after 18 μs simulation time. As shown in Fig. 2, the DAs slowly start to cluster over the 18 μs timeframe, however they do not penetrate or disturb the membrane. 54 ‘mCPT-buSS-Tau’ molecules (39.3 mM) are placed initially randomly above and below a model POPC (1-palmitoyl-2-oleoyl-*sn-glycero*-3-phosphocholine) membrane. Notably, this concentration is about 80-fold higher than the experimental concentration that is reported by Cui *et al.*^35^ At a concentration of 0.05 mM, ‘mCPT-buSS-Tau’ forms nanofilaments with a 6.7 nm width in solution.^35^ In Fig. 2A–F, snapshots show that ‘mCPT-buSS-Tau’ nanoclusters form at the early state of the simulation (within the first 0.01 μs) and persist over the course of the simulation. It is known that the π–π interaction of the aromatic rings on CPT dictate the early nucleation of the DA nanoclusters, according to Kang *et al.*^44^ We quantify the average size of nanoclusters as 
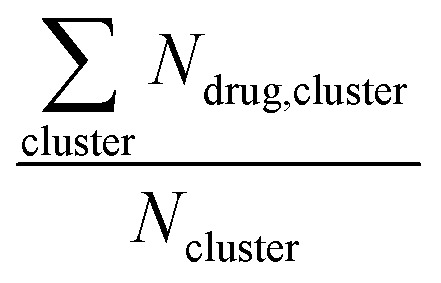
. However, ‘mCPT-buSS-Tau’ contains an average of only five drugs per nanocluster, as shown in Fig. 3 A. Moreover, the distribution of sizes of ‘mCPT-buSS-Tau’ nanoclusters is persistently lower than ‘qCPT-buSS-Tau’ as shown in ESI Fig. 1.† In other words, ‘mCPT-buSS-Tau’ prefers to stay in smaller size nanoclusters. ESI Fig. 1A and B† shows an average of 10 ‘CPTs’ per nanocluster. Furthermore, we also observe that the CPT nanoclusters gradually reach an equilibrium size after 6 μs, with an average of ∼10–15 monomers in 3–6 nanoclusters. At 6 and 13 μs, we observe the formation of a ‘percolated’ nanocluster with all 54 CPTs, but it is not stable and dissolves into smaller size nanoclusters. We define the normalized cluster size as a fraction between 0 and 1 as 
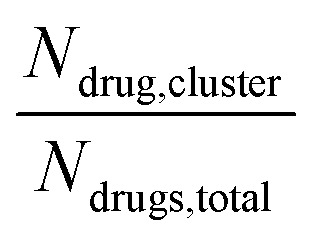
. We group the DA molecules that stay within the *r*_cut-off_ of 4.5 Å. To quantify the shape of each defined nanocluster, we align each nanocluster's principal axes along *x*, *y*, and *z* direction. The radius of gyration, *R*, is derived from the principal moment of inertia, *I* = *mR*^2^, along one direction of each nanocluster where *m* is the total mass in grams of each nanocluster. The ratio of minimum radius of gyration, *R*_min_, to the maximum radius of gyration, *R*_max_, was used to quantify the shape of each nanocluster as the sphericity index, 
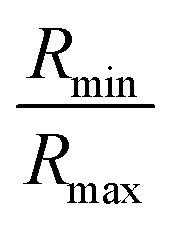
. Using this index, a spherical aggregate will have a value close to 1. In Fig. 3B, small to medium nanoclusters, which corresponds to ∼0.25 normalized size, have a sphericity index of 0.5. This indicates that small nanoclusters are almost ellipsoidal. Interestingly, larger nanoclusters, which reach a normalized size of 0.75, elongate horizontally. This trend is predicted to hold for even larger nanoclusters, since the linear regression shows a negative slope. The anisoptropic growth of ‘mCPT-buSS-Tau’ nanoclusters is supported by Cheetham *et al.*^35^ and Kang *et al.*^44^ studies.

**Fig. 4:**
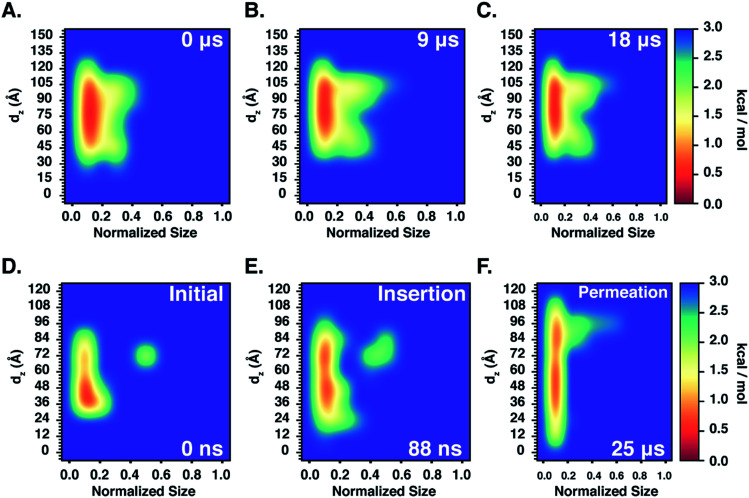
2D Potential of Mean Force (or PMF) profiles that shows the relationship between normalized nanocluster size and the relative distance, *d*_z_, between the lowest points of ‘mCPT-buSS-Tau’ ((A–C) 0–18 μs) and ‘qCPT-buSS-Tau’ ((D–F) 0–25 μs) nanoclusters and the center of mass of the model POPC membrane.

Furthermore, we also characterize the 2D free energy surface that correlates the ‘mCPT-buSS-Tau’ nanocluster sizes and the relative distance, *d*_z_, from the center of mass (COM) of the POPC membrane to the closest point (or closest atom within each nanocluster) of each of the nanoclusters. 2D Potential of Mean Force (PMF). The free energy is derived from the distribution probability using the following equation, 
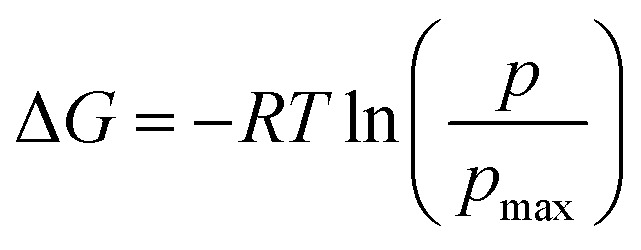
 where *R* is the gas law constant, temperature *T* is in Kelvin and 
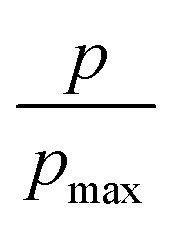
 is the ratio between the probability of a single event to the highest probability event. In Fig. 4A, small to medium ‘mCPT-buSS-Tau’ nanoclusters (∼0.1–0.4 normalized nanocluster size) are placed randomly at least 20 Å above the POPC membrane. Over the course of 18 μs, those small to medium nanoclusters move further away from the membrane as shown in Fig. 4B and C. The free energy minimum is located around 75 to 90 Å with the closest *d*_z_ at 30 Å away. Closer observations show that there are two peaks along the normalized size direction corresponding to two distinctive *d*_z'_s. The first peak with maximum normalized size at 0.4 always stays closer to the membrane, at ∼45 Å, while the second peak with maximum normalized size at 0.6 is staying further away from the membrane, at ∼105 Å. Although the overall normalized size mostly remains the same for the two peaks, the trend indicates that small to medium nanoclusters tend to get closer to the membrane. We hypothesize that the positively charged lysines, on the amphiphilic peptides, when aggregating in larger groups will repulse the positively charged choline head groups of the membrane. This repulsion can hinder the normalized size of the nanocluster and may also prevent their approach to the membrane surface. This could also correlate to the lowest *in vitro* efficiency of ‘mCPT-buSS-Tau’ in cell culture.^35^ However, a definitive answer requires more insightful studies.

### Higher drug loading and interaction with a POPC membrane

**Fig. 5:**
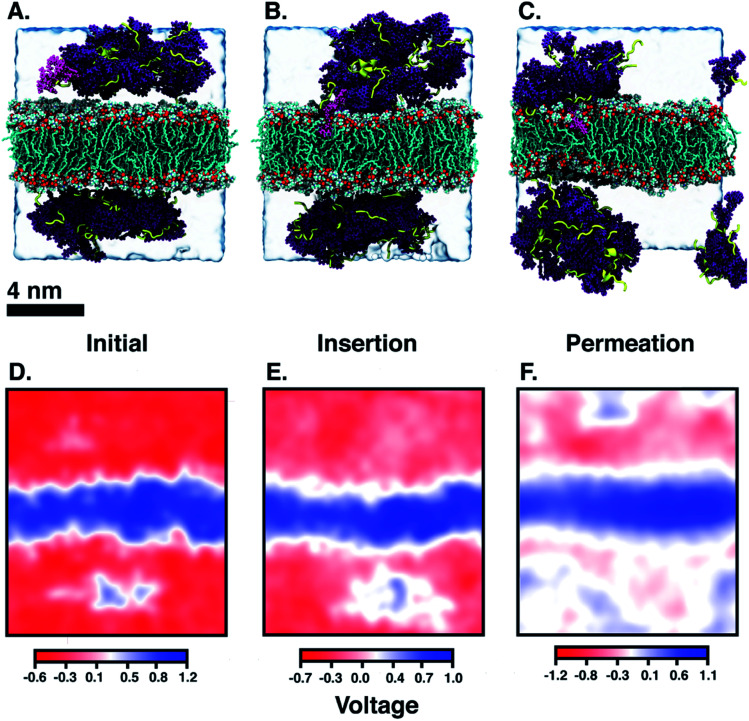
(A–C) Snapshots show ‘nanoclusters’ of ‘qCPT-buSS-Tau’ interacting with the model POPC membrane at the (A) initial (0 ns), (B) insertion (88 ns), and (C) permeation (25 μs) timepoints. Camtothecin (CPT) are in purple with Licorice presentation, peptides are yellow with secondary structure presentation. POPCs are shown in default colors (cyan, red, white) in ‘Licorice’ representation. Water is represented by a transparent box. CPT that inserts itself in the outer hydrophobic core of the membrane is highlighted in magenta. (D–F) 2D electrostatic potential maps after the initial (0 ns), insertion (88 ns), and permeation (25 μs) timepoints demonstrating the positively charged surface of the POPC membrane. The +ve potential and −ve potential are shown in blue and red respectively.

Another DA is ‘qCPT-buSS-Tau’ (Fig. 1 B), which carries four times the number of drugs per peptide (four times the drug loading). However, in this case, due to the increased relative accessibility of the CPT, the drug starts to interact with and penetrate the membrane just after 88 ns (Fig. 5B). After 25 μs we see that the drug builds up in a stacking configuration in the outer hydrophobic core of the membrane, starting to bend the membrane, pulling the positively charged peptide groups towards the membrane (Fig. 5C). The electrostatic potential at and surrounding the membrane, before contact with the drugs, during initial drug penetration of the membrane, and after 25 μs, is also shown in Fig. 5D–F. 54 qCPT molecules or 54.7 mM are placed randomly above and under the POPC membrane, same as previously described for the ‘mCPT-buSS-Tau’-membrane system. Again, this concentration is approximately 80-fold higher than the concentration that forms 9.5 nm-width nanotubes in TEM and cryo-TEM experiments.^35^ Over a 25 μs simulation, we find more significant interaction of the ‘qCPT-buSS-Tau’ with the model POPC membrane than with the ‘mCPT-buSS-Tau’ nanoclusters. Indeed, we find that the ‘qCPT-buSS-Tau’ nanoclusters penetrate and insert in the membrane at ∼100 ns with the drugs eventually stacking up at the membrane surface and permeating the membrane as shown in Fig. 5. Here, we identify two critical events: *insertion* at 88 ns and deeper partitioning of the drug into the membrane or *permeation* at 25 μs. In contrast to ‘mCPT-buSS-Tau’, after 6 μs, we do not observe an equilibrium state of the ‘qCPT-buSS-Tau’ nanoclusters, in terms of the average size or number of molecules per cluster, as shown in ESI Fig. 1C and D.† Indeed, the average size still decreases after 25 μs as the number of nanoclusters is increasing. After the insertion event at 88 ns, the original larger nanoclusters dissolve into smaller nanoclusters which is shown as a decrease of the average size from 10 to 6 in Fig. 3A. Also, the sphericity, which describes the overall shapes of the ‘qCPT-buSS-Tau’ nanoclusters, follows a different trend compared to ‘mCPT-buSS-Tau’.

As shown in Fig. 3B, small to medium nanoclusters of ‘qCPT-buSS-Tau’ have a similar sphericity index of ∼0.5 as for the ‘mCPT-buSS-Tau’. In other words, the overall shape of the small to medium drug-amphiphilic peptides are ellipsoidal regardless of their chemical structures. Nevertheless, when qCPT nanoclusters reach more than ∼0.5 normalized size, their sphericity index rapidly approaches 1.0, indicated they become rounder/more spherical. A positive linear regression predicts this trend to be followed when a normalized size of 1 is reached. We postulate that such a spherical shape in larger qCPT nanoclusters may nucleate nanotube formation, which is observed in experiments.^35^ However, it is unclear what the role of a membrane interface may play in facilitating self-assembly.

Next, we characterize the 2D free energy surface to investigate the relationship between the ‘qCPT-buSS-Tau’ nanocluster sizes and the relative distance, *d*_z_, between the nanocluster and the COM of the POPC bilayer. As mentioned, *d*_z_ measures from the lowest point of each nanocluster (or closest atom to the membrane in the nanocluster) to the COM of the membrane to characterize the penetration, or closest point of approach, of the drugs. From Fig. 4D, we observe two ‘qCPT-buSS-Tau’ nanocluster populations: the first dominant population with ∼0.1 normalized size (small to medium sizes), and the second population with ∼0.5 normalized size (larger sizes). Initially, the first population is concentrated mostly at ∼36 Å (0 kcal mol^−1^) and at ∼72 Å (1 kcal mol^−1^) with its minimum *d*_z_ is at 24 Å while the second population is located at ∼72 Å (∼2.5 kcal mol^−1^). Moreover, ‘qCPT-buSS-Tau’ nanoclusters are 12 Å closer to the membrane compared to ‘mCPT-buSS-Tau’. One may argue that the initial random configurations favor a closer distance for ‘qCPT-buSS-Tau’ than ‘mCPT-buSS-Tau’, as shown in Fig. 2A and 5A, respectively. Nevertheless, the ‘Insertion’ event at 88 ns has the minimum *d*_z_ of the first population of drugs that penetrate the membrane as deep as 12 Å; and at the end of the simulation, the penetration is at its deepest, which is only 6 Å away from the COM of the membrane, as shown in as shown in Fig. 4E and F. Visual inspection of Fig. 5B shows that only 8 CPT drugs, colored in magenta, diffuse past the outer head group layer. While there is clear membrane bending after 25 μs simulation (Fig. 5C), the local membrane thickness and the *x*–*y* positions of the drugs that position themselves in the outer hydrophobic core of the membrane are only weakly correlated, as shown in ESI Fig. 2.†

As mentioned, ‘qCPT-buSS-Tau’ nanoclusters break into smaller sizes after insertion but their clustering propensity is still high. In other words, after breaking apart, ‘qCPT-buSS-Tau’ nanoclusters reorganize and form aggregates again. We hypothesize that there are competing free energy contributions between the *inter*- and *intra*-nanocluster interactions. In other words, the enthalpic interactions between the POPC and the ‘qCPT-buSS-Tau’ nanoclusters, specifically the camptothecin,^43^ are more favorable than the mainly hydrophobic and stacking interactions that hold the ‘qCPT-buSS-Tau’ nanoclusters together. As a result, the POPC membrane allows the ‘qCPT-buSS-Tau’ nanoclusters inside, leading *via* the hydrophobic CPT, breaking the larger aggregates.

When the DAs insert into the membrane, drug first, we hypothesize that the insertion may affect the overall electrostatics at the membrane interface. Since the peptides contain multiple positively charged lysines, we expect to see a shift in the electrostatic potential along the *z*-direction of the membrane, as the peptides are pulled closer to the membrane surface. The choline and phosphate phospholipid head groups at the interface give the membrane surface a net dipole moment, with the positively charged cholines on the surface. Fig. 5D–F shows the electrostatic potential (EP) map that is calculated from the PMEPot^68^ plugin in Visual Molecular Dynamics (VMD)^67^ using fast Fourier transformations to solve Poisson's equation numerically. A detailed explanation can be found in Aksimentiev *et al.*^68^ Only a slice through the simulation box at the insertion site above and below the membrane surrounding the CPT drugs are shown. We find that the membrane is more positive than the surrounding water, which contains Cl^−^ ions and is net negatively charged. The hydrophobic CPT drugs are buried inside the amphiphilic peptides. In the initial configuration, there is a net positive region (0.1–0.5 V) that appears in the surrounding water. This is attributed to the positively charged lysines, that are mostly buried. However, after 25 μs, more lysines are pointing outwards as multiple more positive regions (∼1 V) appear around the membrane in the surrounding water. However, this would in effect put a positively charged ‘coat’ on the qCPT nanoclusters. As a result, the net positive charge of the membrane surface may repulse this positively charged ‘coat’ on the qCPT nanoclusters. The interaction between the membrane surface and larger self-assemblies will be quantified in more detail in the next section.

**Fig. 6:**
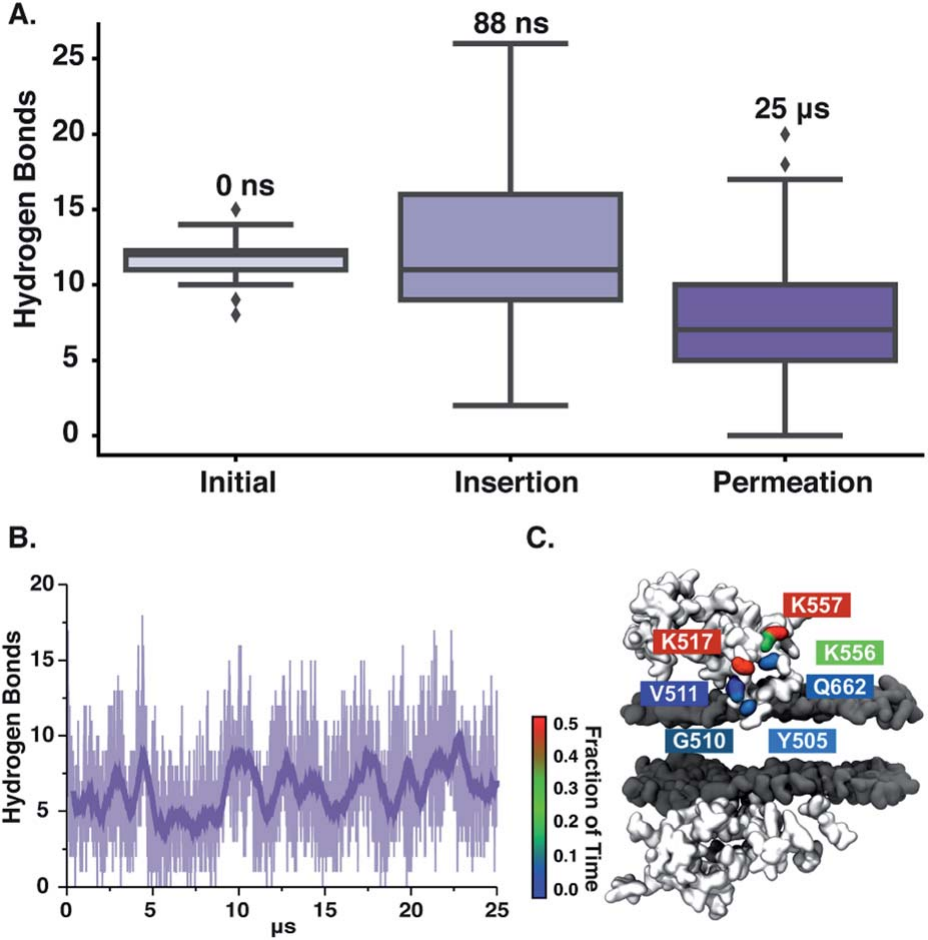
(A) Total hydrogen bonds after the initial (0 ns), insertion (88 ns), and permeation (25 μs) timepoints. The lowest, middle, and top lines of the boxes are 25, 50, and 75 percentiles, respectively. The top and bottom lines are maxima and minima, with the outliers as black diamonds (B). The average hydrogen bonds between ‘qCPT-buSS-Tau’ and the POPC bilayer during 25 μs trajectory. The transparent line shows the raw data and the opaque line shown the running average over the previous 100 frames. (C) The amino acid involved and the lifetime of the associated hydrogen bonds between nanoclusters of the ‘qCPT-buSS-Tau’ and the POPC membrane.

We next observe that ‘qCPT-buSS-Tau’ nanoclusters anchor themselves above the membrane, while ‘mCPT-buSS-Tau’ nanoclusters fail to do so. Hydrogen bonds between the peptides and the membrane surface may play a crucial role in this anchoring step. In Fig. 6B, an average of 5–10 hydrogen bonds persistently exist between ‘qCPT-buSS-Tau’ nanoclusters and the membrane, while there is an average of only one hydrogen bond between ‘mCPT-buSS-Tau’ and the membrane (as shown in ESI Fig. 3†). Clearly, the multi-fold difference in hydrogen bonds between mCPT and qCPT reemphasizes the important role of the hydrogen bonds in forming an anchor for the nanoclusters above the membrane surface. Additionally, Fig. 6A shows a significant increase in hydrogen bonds formed during insertion, most of them lasting 25 μs. As the result, we calculate the lifetimes of hydrogen bonds between ‘qCPT-buSS-Tau’ nanoclusters and the POPC membrane. Hydrogen bond analysis was performed using the Cpptraj package^69^ from AmberTools package. The cut-off angle between the hydrogen donor-hydrogen atom-hydrogen acceptor was 120°, and the cut-off distance between hydrogen donor-hydrogen acceptor was 3 Å. We considered all atoms on ‘qCPT-buSS-Tau’ and POPCs. In Fig. 6C, K517, K556, K557 have the most extended lifetimes spanning 30–50% of the 25 μs trajectory. G510 occurs during 10% of the trajectory. V511, Q662, Y505 are the least significant. K517 and G510 are on the same DA. K556 and K557 are on the same DA. V511, Q662, and Y505 are on different DAs. Both K517 and K557 are outer lysines in the peptide sequence. The rest of the residues, which are not shown, form extremely short lifetime hydrogen bonds with the membrane. Surprisingly, we find a high density of persistent hydrogen bonds near the insertion site between the hydrophobic CPT and the membrane. We hypothesize that ‘qCPT-buSS-Tau’ nanoclusters initially form scattered stable hydrogen bonds with the POPC membrane surface, effectively anchoring the nanoclusters to the surface of the membrane. Then, more stable hydrogen bonds aggregate, which can facilitate a high-density hydrogen bonding region on the surface of the nanoclusters. Eventually, the free energy tips such that it becomes more favorable to break the nanoclusters apart, following by the reformation of such nanoclusters as several molecules leave and insert themselves in the membrane.

### Umbrella sampling

**Fig. 7:**
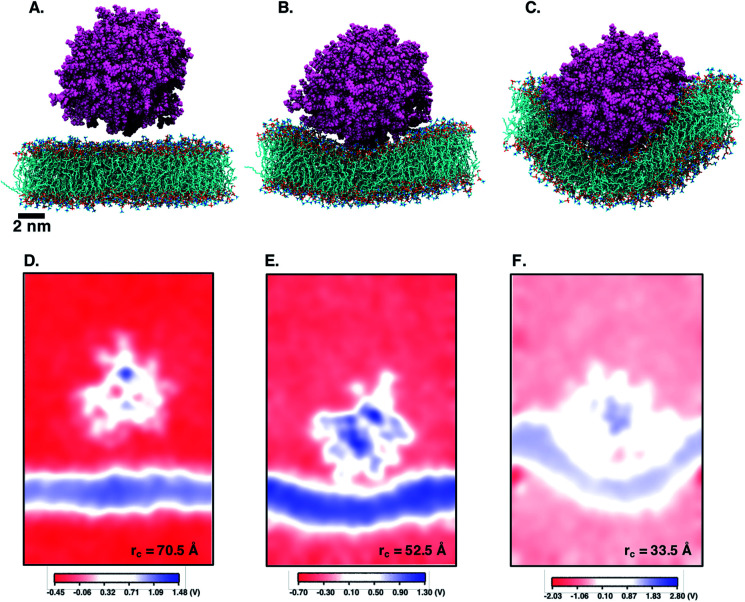
Snapshots from umbrella sampling calculations showing nanofilament interaction with the membrane at three reaction coordinates (*r*_c_) selected when nanofilament is (A) away from the membrane (*r*_c_ = 70.5 Å) (B) approaching the membrane (*r*_c_ = 52.5 Å) and (C) in contact with the membrane (*r*_c_ = 33.5 Å). 2D electrostatic potential maps for nanofilament interaction with the membrane when nanofilament is (D) away from the membrane (E) approaching the membrane and (F) in contact with the membrane. The +ve potential and −ve potential are shown in blue and red respectively.

**Fig. 8:**
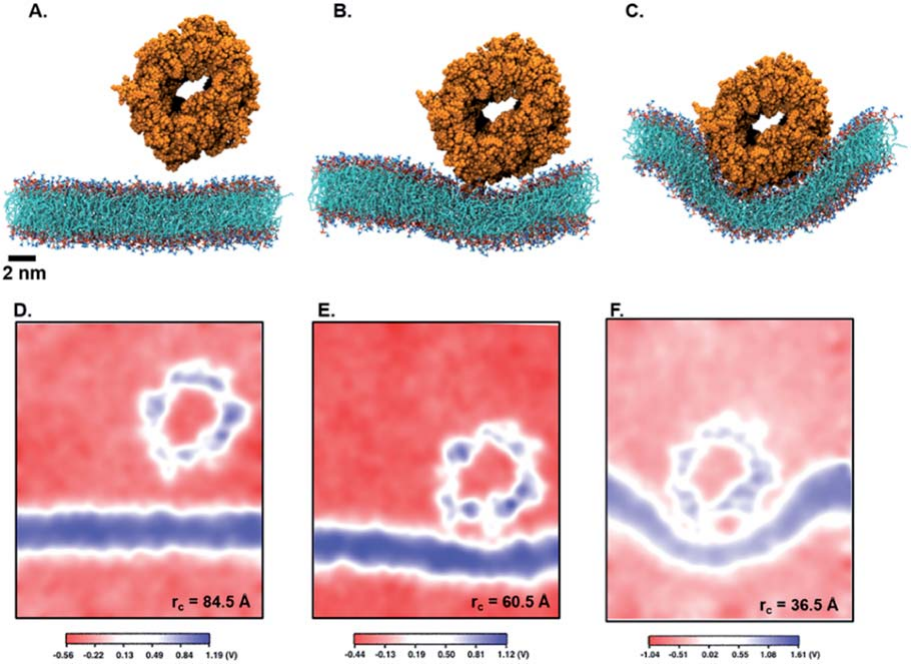
Snapshots from umbrella sampling calculations showing the nanotube interaction with the membrane at three reaction coordinates (*r*_c_) selected when nanofilament is (A) away from the membrane (*r*_c_ = 84.5 Å) (B) approaching the membrane (*r*_c_ = 60.5 Å) and (C) in contact with the membrane (*r*_c_ = 36.5 Å). Electrostatic potential maps for nanotube interaction with the membrane when nanotube is (D) away from the membrane (E) approaching the membrane and (F) in contact with the membrane. The +ve potential and −ve potential are shown in blue and red respectively.

Next, we perform umbrella sampling (US) to determine the interaction energy of pre-assembled ‘mCPT-buSS-Tau’ nanofilaments and ‘qCPT-buSS-Tau’ nanotubes with the POPC model membrane. Fig. 7A, B and C shows snapshots of three windows from the US of the nanofilament interaction with the POPC membrane for reaction coordinates (*r*_c_) of 70.5 Å, 52.5 Å and 33.5 Å respectively. These three snapshots represent three events during US – when the nanofilament is away from the membrane, approaching the membrane, and in contact with the membrane. These snapshots clearly indicate the changing structure of the membrane as the nanofilament approaches it – initially the membrane bends and then wraps the nanofilament. We do not observe any penetration of the membrane by the nanofilament suggesting a strong repulsive interaction between the membrane and the nanofilament. Next, we look at the electrostatic potential (EP) map during the three events mentioned above (Fig. 7C–E). The EP map is calculated in VMD^67^ for the last 10 ns of the 28 ns simulation of each US window. In these maps, we observe the solvent is negatively charged (red), the nanofilament and the membrane are both positively charged (blue) and the surrounding water solution is neutral (white). The nanofilament is positively charged with protonated lysines surrounding the periphery of the nanofilament. The system has been neutralized by Cl^−^ ions. Thus, it makes the solvent more negatively charged compared to the membrane and the nanofilament. The POPC membrane is composed of zwitterionic phosphatidylcholines and we observe the membrane is positively charged compared to its surroundings. The electrostatic potential maps for three events shift as the nanofilament approach the membrane. We find that the nanofilament is most positive when it is approaching the membrane (Fig. 7E) compared to when the filament is away (Fig. 7D) or in contact (Fig. 7F) with the membrane. Similarly, Fig. 8 shows the snapshots of the ‘qCPT-buSS-Tau’ nanotube interaction with the membrane and the corresponding EP maps. The *r*_c_'s of the three events when the nanotube is away from the membrane, approaching the membrane, and in contact with the membrane are 84.5 Å, 60.5 Å and 36.5 Å respectively (Fig. 8A, B and C). Similar to the nanofilament scenario, we find that as the nanotube approaches, the membrane starts bending and then wraps the nanotube. Furthermore, the EP maps of the nanotube interaction with the membrane is complementary to the trend of nanofilament interaction with the membrane. We find the nanotube and the membrane is positively charged (blue) and the solvent is negatively charged (Fig. 8D–F).

**Fig. 9:**
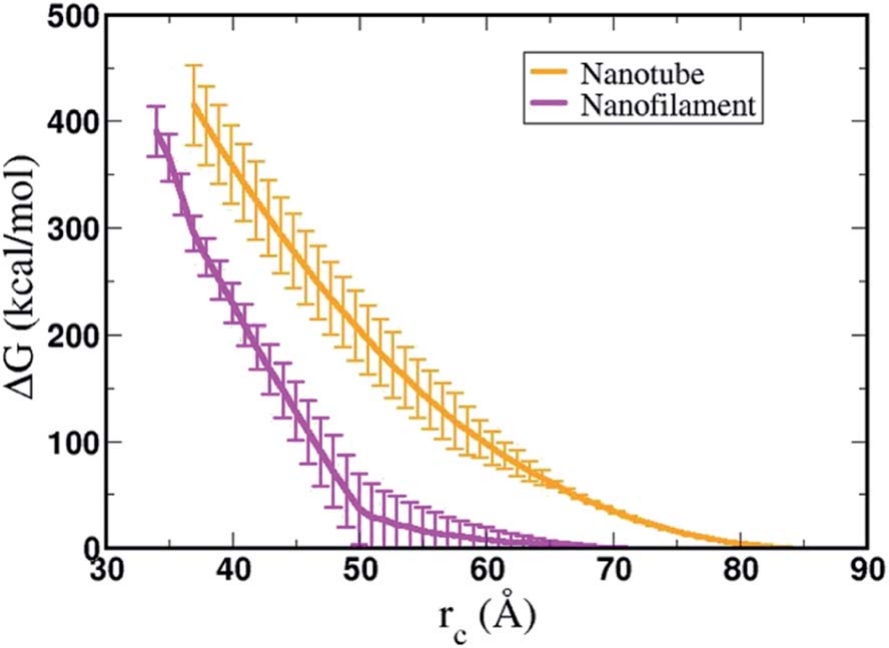
Free energy profile, Δ*G* (kcal mol^−1^), calculated by umbrella sampling for the interaction of nanofilament (magenta) and nanotube (orange) with the POPC model membrane. The reaction coordinate (*r*_c_) is the difference between center of mass (COM) of the membrane and the nanotube or the nanofilament, respectively.

**Fig. 10:**
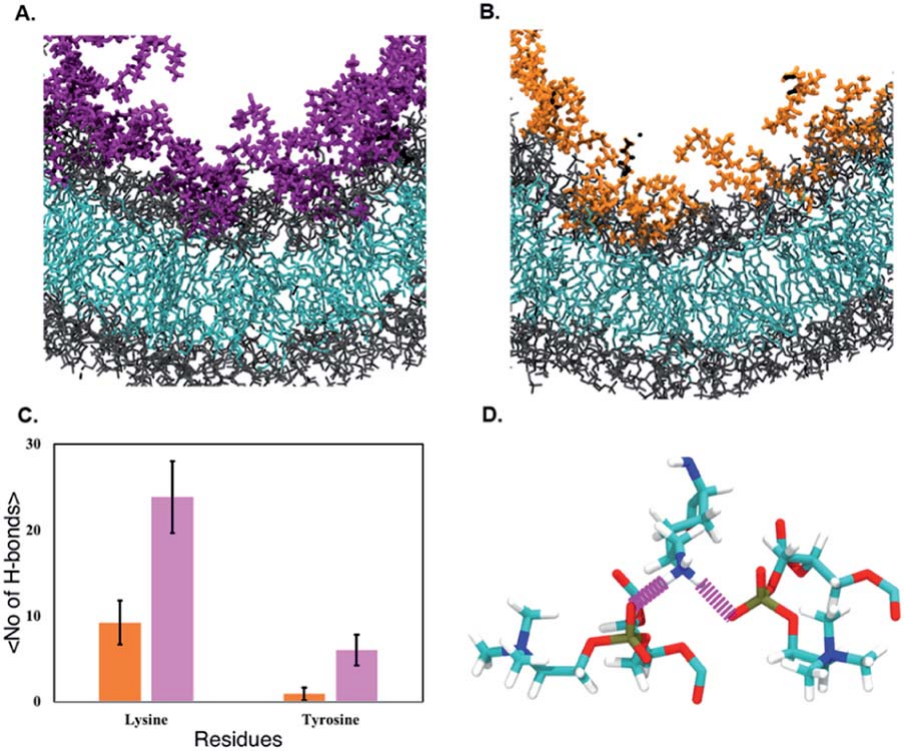
Interaction of lysines of the (A) nanofilament (magenta) and (B) nanotube (orange) with model POPC membrane (head groups shown in gray and tail groups shown in cyan). (C) Average no. of H-bonds formed by lysines and tyrosines of the nanofilament (magenta) and the nanotube (orange) with POPC membrane during last 10 ns of 28 ns simulation in the closest umbrella sampling window. (D) Hydrogen bonds between the amine of the lysine and phosphate of the POPC head groups.

Fig. 9 shows the resulting PMF profile determined by US for the interaction of the nanofilament and the nanotube with the membrane. We find similar trends for both systems – the interaction energy increases as the nanofilament/nanotube approaches the membrane. However, it is energetically less costly for the nanofilament compared to the nanotube to interact with the membrane. This suggests that the nanofilament succeeds in making a higher number of favorable interactions with the membrane compared to the nanotube with the membrane. We find that hydrogen bonds between the nanofilament/nanotube with the membrane is one of the major intermolecular interactions. Each DA has two lysines which forms the outer periphery of the nanofilament and the nanotube, creating direct contact between lysines and the membrane (Fig. 10A and B). Since the nanofilament has a smaller radius ∼4.5 nm compared to the nanotube of radius ∼5.0 nm, the outer surface area per 10 nm length for the nanofilament ∼283 nm^2^ is smaller than the surface area of the nanotube ∼321 nm^2^. Thus, lysines are packed much more closely for the nanofilament than for the nanotube. The density profile (Fig. S4†) of the last window from US further indicates that the phosphate head groups of the membrane and the lysines of the nanofilament and the nanotube are overlapping. Using VMD, we calculate the number of hydrogen bonds between the nanofilament and the nanotube with the membrane for two events – when approaching, and when in contact with the membrane. As shown in Fig. S5,† the average number of hydrogen bonds increases as the nanofilament/nanotube approaches and then contacts the membrane. For both events, the average number of hydrogen bonds is significantly greater for the nanofilament–membrane interaction compared to nanotube–membrane interaction. Next, we characterize the residues from the nanofilament/nanotube forming hydrogen bonds with the membrane. For both systems, we find the number of hydrogen bonds for different residues of the nanofilament/nanotube with the membrane is directly related to their position. As shown in Fig. 10C, the average number of hydrogen bonds between lysines of the nanofilament and the nanotube with the membrane is significantly higher compared to tyrosines. Lysines being at the outer periphery of the nanofilament and the nanotube have direct contact with the membrane resulting in higher hydrogen bonds between lysines and the membrane. Tyrosine is the next inner residue after lysines in the DAs. Since tyrosines have less access to the membrane compared to lysines, the average number of hydrogen bonds between tyrosines and the membrane is significantly lower compared to the hydrogen bonds between lysines and the membrane. The fourth inner residue valine has negligible (∼1) hydrogen bonds with the membrane for both systems. In Fig. 10D, we show the representative hydrogen bonds between lysine and membrane phosphate head groups. The greater hydrogen bonds of the nanofilament–membrane compared to nanotube–membrane supports the lower PMF for the nanofilament–membrane interaction compared to the nanotube–membrane interaction.

**Fig. 11:**
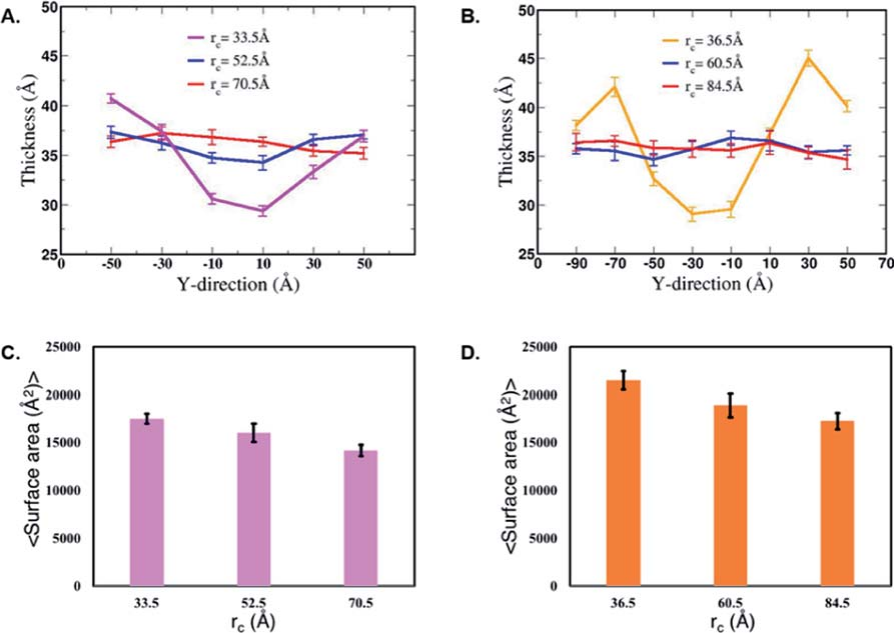
Thickness of the membrane during interaction with the (A) nanofilament and (B) nanotube. Average surface area of the membrane during interaction with the (C) nanofilament and (D) nanotube. These values are calculated for the last 10 ns of the 28 ns simulation of the selected umbrella sampling windows. The thickness and average surface area for these systems are calculated at three timepoints – when the nanofilament/nanotube is away from the membrane, approaching membrane, and in contact with the membrane with decreasing *r*_c_ values as shown above.

Next, we characterize structural changes in the membrane as the nanofilament/nanotube approaches the membrane surface. Fig. 7 and 8 clearly show the membrane bending as the nanofilament/nanotube approaches. We calculate the change in thickness and surface area of the membrane during the three events previously described. The calculations are discussed in methods section. We find that the thickness of the membrane along the *Y*-axis decreases as the nanofilament/nanotube approaches and makes contact with the membrane. Fig. 11A and B shows the original membrane thickness of ∼38 Å when the nanofilament/nanotube is far away from the membrane, which significantly decreases to the thickness of ∼28 Å when the nanofilament/nanotube makes contact with the membrane. The change in thickness is dominant where the nanofilament/nanotube is in direct contact with the membrane (∼10 Å thinner). Next, we calculate the surface area of the membrane during these three events (Fig. 11C and D). We find the surface area of the membrane is greater for the membrane when in contact with the nanofilament/nanotube compared to when not in contact with the nanofilament/nanotube, with an increase of ∼2000 Å^2^. The difference for the thickness and the surface area of the membrane is most significant between two end events – the nanofilament/nanotube away from the membrane and the nanofilament/nanotube in contact with the membrane. For the inner window, when the nanofilament/nanotube is approaching the membrane, the values of the membrane thickness are similar to the corresponding values when nanotube/nanofilament is further away from the membrane, while the surface area of the membrane has already increased. This suggests that the membrane first bends, without any contact with the nanofilament/nanotube, and then only thins upon contact.

Overall, using umbrella sampling, here we determine the interaction free energy of DA nanofilament and nanotube with a model POPC cell membrane. Membrane bending and wrapping of nanostructures of varying shapes has been characterized by coarse grained simulations of spherical nanoparticles and spherocylinders by Frenkel *et al.*^70^ It is found that prolate shapes can lead to more efficient delivery. Here we find the interaction energy between the nanostructures and the membrane is very high (>400 kcal mol^−1^), suggesting that the process is energetically costly, but more costly for a nanotube *vs.* a nanofilament. We assume the bending rigidity *k*_c_ for a model POPC membrane and estimate the bending energy of the membrane, 
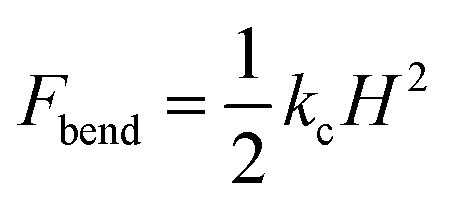
, where *H* is the mean curvature of the membrane. The bending energy of the membrane, *F*_bend_, costs ∼1/3 of the total interaction energy (∼130 kcal mol^−1^ for the nanofilament system, and ∼100 kcal mol^−1^ for the nanotube system), as described in the methods section. Previous computational studies by Liu *et al.*^71^ have reported that clathrin-mediated endocytosis is easier for a softer membrane than a rigid membrane. These studies, conducted with varying membrane rigidity, found it is difficult for a rigid membrane to deform and form the vesicle necessary for endocytosis.^71^ However, this is a simplistic representation of the system and composition of the membrane as compared with a realistic membrane composition of the plasma membrane of the cancer cell. Notably, molecular dynamics simulations are moving towards more realistic phospholipid compositions to mimic real cells.^72^ Significant differences have been observed in the interaction of cationic nanoparticles with zwitterionic *vs.* charged membranes. Computational studies by Cui *et al.*^73^ have reported that cationic gold nanoparticles have lower affinity to zwitterionic membranes but readily bind to 9 : 1 zwitterionic:anionic model membranes.^73^ Thus, including a range of anionically charged phosphatidylserine (PS), to mimic the negative charge that is found in cancerous cells^74^ may significantly modulate the degree of repulsion.

To summarize, the high interaction free energy, electrostatic potential maps and the bending phenomena of the membrane indicate strong repulsive interaction between the nanostructures and the membrane. We find the interaction free energy of the nanofilament and the membrane is lower than the interaction free energy of the nanotube and the membrane. We suggest that one of the factors contributing to this difference is the number of hydrogen bonds each nanostructure makes with the membrane. The nanofilament, having smaller radius than the nanotube, has a higher lysine density on its surface facilitating more hydrogen bonds between the nanofilament and the membrane. During the simulations we did not observe pore formation in the membrane, nor significant deformation of the nanostructures. This suggests these nanostructures are very stable structures and to break these nanostructures into DAs will be energetically costly. Previous all-atomistic and coarse-grained simulations of these anticancer nanostructures have shown these structures remain intact throughout simulations (∼microseconds), stabilized by π–π interactions between the DAs.^44,52,75,76^ Thus, nanostructures will not dissemble and release DAs and rather interact and traverse through the membrane as a one entity. Our observation of membrane bending and wrapping of the nanostructures suggest endocytosis as the possible mechanism for the internalization of these nanostructures by the membrane.

## Conclusions

Here, we report long-time molecular dynamics simulations (up to 25 μs) to characterize how the DA structure affects the translocation across and interaction with the cellular membrane. Briefly, we find that DAs with lower drug loading (one drug attached per peptide) do not interact with or penetrate the membrane after very long timescales (18 μs). The results for a slightly different structure of the DA, four hydrophobic drugs attached per peptide, instead of only one drug attached, are strikingly different. In this case, due to the increased relative accessibility of the CPT, the hydrophobic cancer drug starts to interact with and penetrate the membrane after only 0.5 μs. After 25 μs we see that the drug builds up in a stacking configuration in the outer hydrophobic core of the membrane, starting to bend the membrane, pulling the positively charged peptide groups towards the membrane surface. Thus, singular DAs are suggested to interact with the membrane *via* a simple diffusive mechanism at shorter time-scales (<1 microsecond), but, at longer timescales (≥10 μs) a more active mechanism. Next, using advanced sampling methods in molecular dynamics, we determine the potential energy of interaction of varying DA nanostructures—both nanofilament and nanotube—with the cell membrane. Here we find that both nanostructures repel the membrane due to their high positive surface charge density; however, the nanotube, with its increased diameter, is more repulsive. The membrane thins and bends as both nanostructures approach the membrane surface. Moreover, we find that hydrogen bonds between the nanostructure and the membrane may display a critical role in mediating membrane permeation. These results suggest that the interaction of engineered peptide sequences with model cellular membranes can be tailored through increasing the relative hydrophobicity of the molecule and through control of the hydrogen bond density between the nanostructure and membrane.

## Conflicts of interest

There are no conflicts to declare.

## Supplementary Material

NA-003-D0NA00697A-s001

NA-003-D0NA00697A-s002

NA-003-D0NA00697A-s003

NA-003-D0NA00697A-s004

NA-003-D0NA00697A-s005

NA-003-D0NA00697A-s006
