# Supplementary material for: The interaction of supramolecular anticancer drug amphiphiles with phospholipid membranes
Source: Nanoscale Adv. 2020 Oct 26;3(2):370–82. doi: 10.1039/d0na00697a (PMC8010983; doi:10.1039/d0na00697a)
Supplement: NA-003-D0NA00697A-s002 [file NA-003-D0NA00697A-s002.pdf]

Electronic Supplementary Material (ESI) for Nanoscale Advances.
This journal is © The Royal Society of Chemistry 2021

The Interaction of Supramolecular Anticancer Drug Amphiphiles with Phospholipid Membranes

Phu K. Tang†‡*, Anjela Manandhar†‡*, William Hu§, Myungshim Kang†, Sharon M. Loverde†‡

† Department of Chemistry, College of Staten Island, City University of New York, 2800 Victory Blvd., 6S-238, Staten Island, NY 10314

‡ Ph.D. Program in Chemistry, Biochemistry, and Physics, The Graduate Center of the City University of New York, New York, United States

§ Hunter College High School, New York, NY 10128

*co-first authors

Email: sharon.loverde@csi.cuny.edu

Supporting Information.

All umbrella sampling calculations were done with the collective variables module[1] in NAMD[2].

Thickness of the membrane. Here, the membrane is divided into blocks of 20 Å along Y-direction. The thickness (T) for each block is calculated as the absolute difference between the average center of mass (COM) of all phosphorus (P) atoms of upper leaflet and lower leaflets.

$$Thickness = \left|COM^{P}_{upper} - COM^{P}_{Lower}\right| \quad \text{[Eq. 1].}$$

Surface area of the membrane. First the length of the curved membrane (L) was calculated as the sum of distance between the consecutive COMs of the phosphorus of upper leaflet for each 20 Å block along Y-direction. Then the surface area of the membrane is calculated as:

$$Surface\ area = Length\ \times Width \qquad \text{[Eq. 2].}$$

Here W is the width of the membrane (dimension of membrane along X-direction).

Surface area of nanostructures. The radius (r) of the nanofilament is ~4.5nm and of the nanotube is ~5.1nm. The surface area (A) of each nanostructure is calculated as:

$$A = 2\pi rh \qquad \text{[Eq. 3].}$$

Thus, for length (h) of 10nm, the surface area of the nanofilament is ~283nm^2 and of the nanotube is ~321nm^2.

Bending energy of the membrane. We calculate the approximate bending energy of the POPC membrane model as:

$$F_{bend} = \frac{1}{2}k_c H^2 \qquad \text{[Eq. 4].}$$

Here, k_c is bending rigidity of the POPC membrane (~14.6 x 10^{-20} J) and H is the mean curvature calculated as:

$$H \sim \frac{1}{r} \qquad \text{[Eq. 5].}$$

We assume the mean curvature of the membrane is determined by the radius of the nanocluster (r). For the nanofilament of radius ~4.5 nm, $F_{bend,f}$ ~ 0.52 kcal/mol.nm^2, and for the nanotube of radius ~5.0nm, $F_{bend,t}$ ~ 0.42 kcal/mol.nm^2.

The total bending energy of the membrane is calculated as:

$$F_{bend\_total} = F_{bend} \text{ x } A_{membrane} \qquad \text{[Eq. 6].}$$

The approximate area of the membrane is estimated as the area of a rectangular patch having the same length and width as that of their equilibrated box. Hence, $A_{membrane}$ of the nanofilament is (16 nm x 15.8 nm) 252.8 nm^2, and of the nanotube is (12.3 nm x 19.6 nm) 241.08 nm^2. Thus, we estimate the total bending energy of the membrane interacting with the nanofilament is $F_{bend,total}$ = 131.23 kcal/mol, and the total bending energy of the membrane interacting with the nanotube is $F_{bend,total}$ = 101.37 kcal/mol.

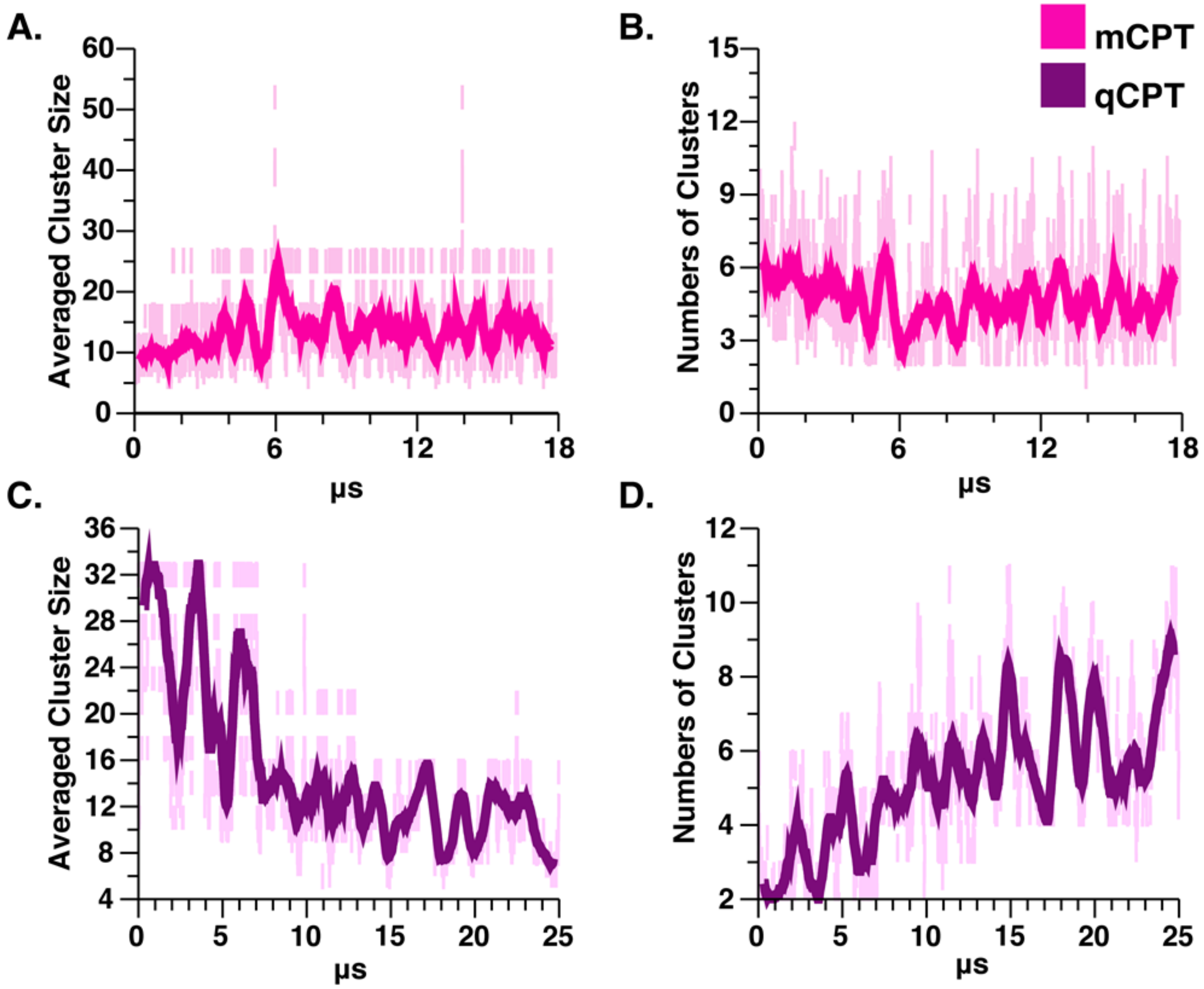

S1. Average cluster sizes and number of clusters for A-B. 'mCPT-buSS-Tau' and C-D. 'qCPT-buSS-Tau'. Average cluster size is the number of hydrophobic drugs (CPTs) per cluster.

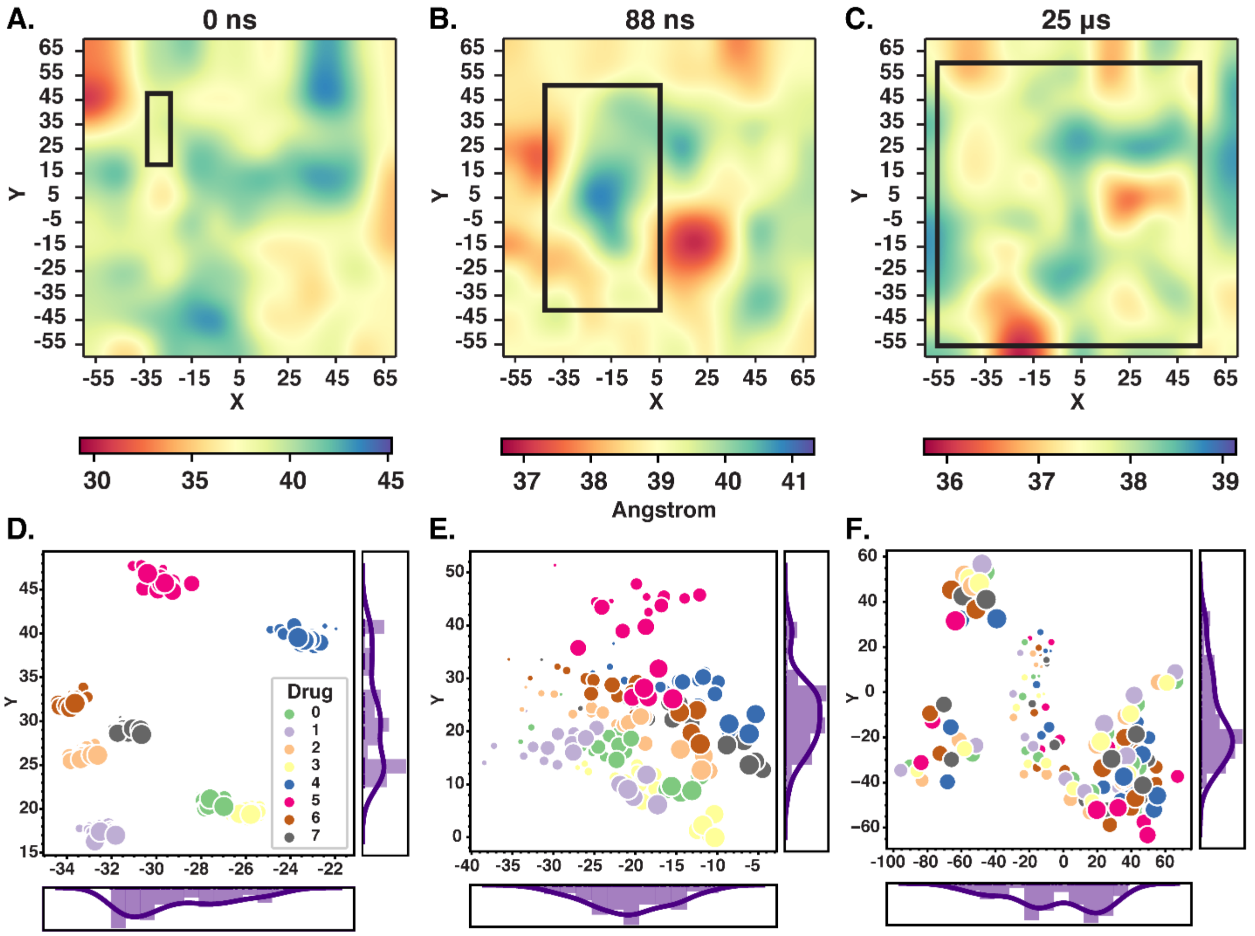

S2. A-C. Local thickness profiles of the POPC membrane at three critical events: Initial (0 ns), Insertion (88 ns) and Permeation (25 microsecond). The black boxes shows the positions of the 8 drug-portions on the thickness profiles. D-F. Tracking positions of 8 drug-portions that penetrate the membrane the deepest. To smooth out the average, only the last 20 frames of each event are considered. Larger size shows ending of the trajectory.

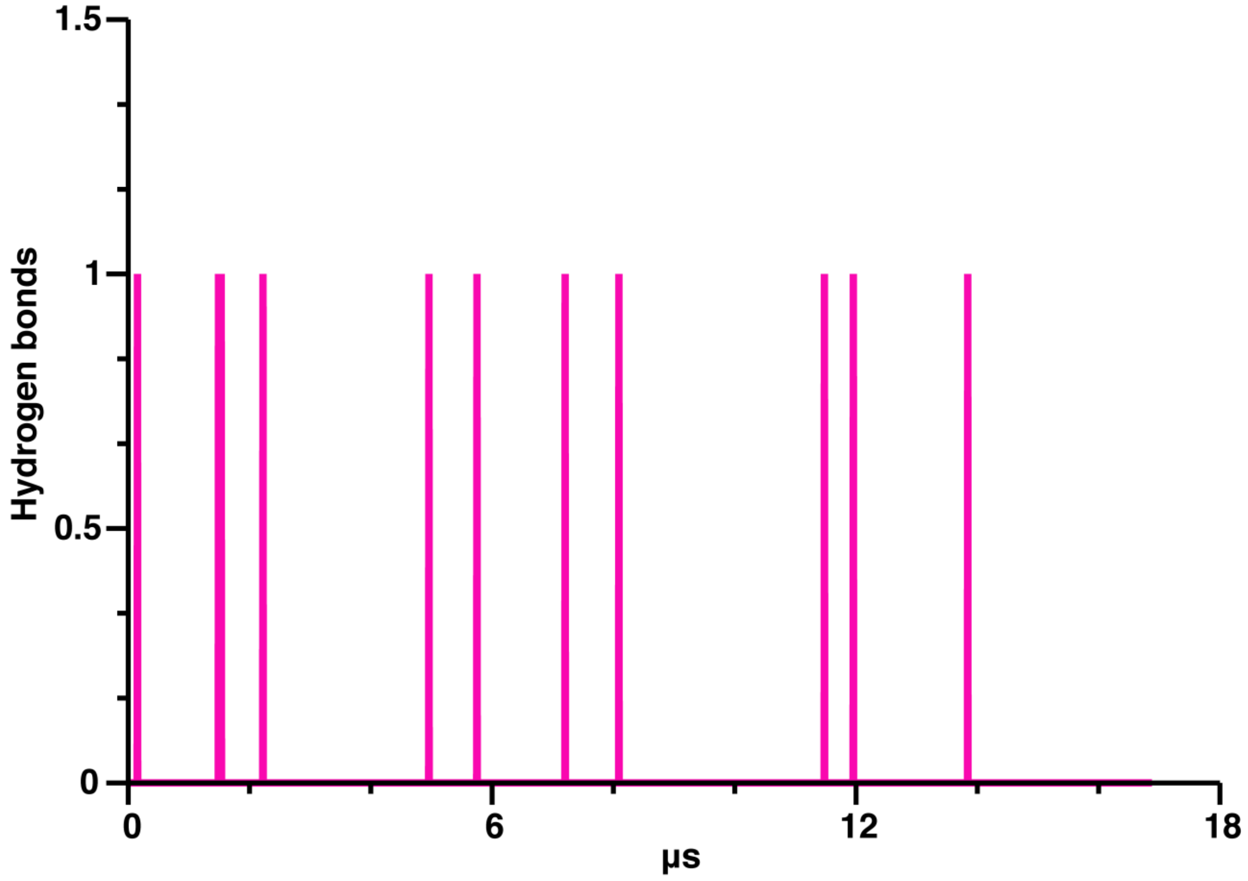

S3. Hydrogen bonds over time in mCPT-buSS-Tau/membrane system

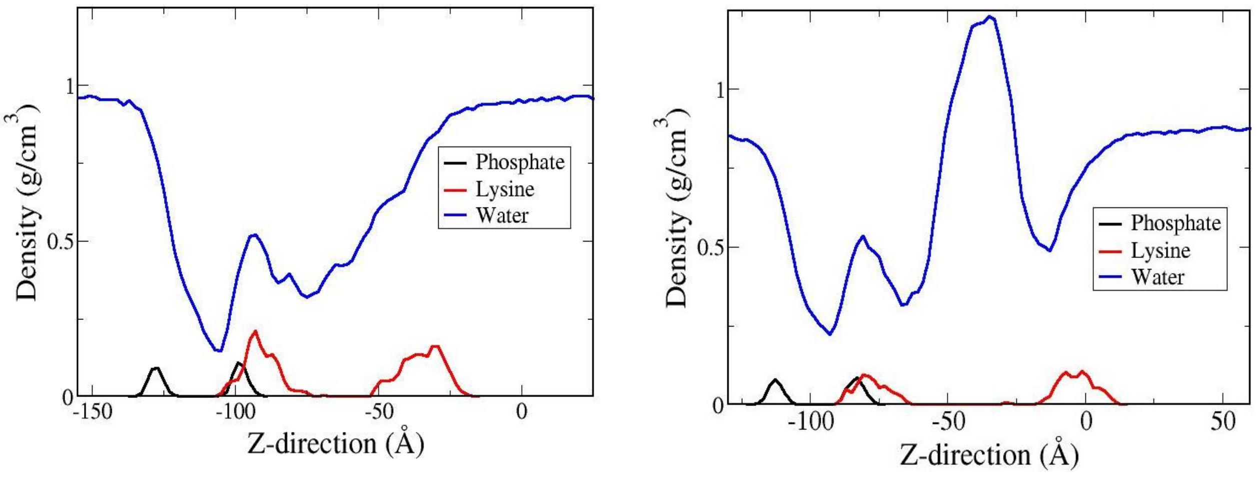

Figure S4. Density of water, lysines of the nanostructures and phosphates of the membrane across Z-direction along the middle section (~30 Å) of **A.** 'mcpt-buSS-Tau' filament **B.** 'qcpt-buSS-Tau' nanotube calculated for the last 10ns of the 28ns simulation when the nanofilament and the nanotube is in contact with the membrane.

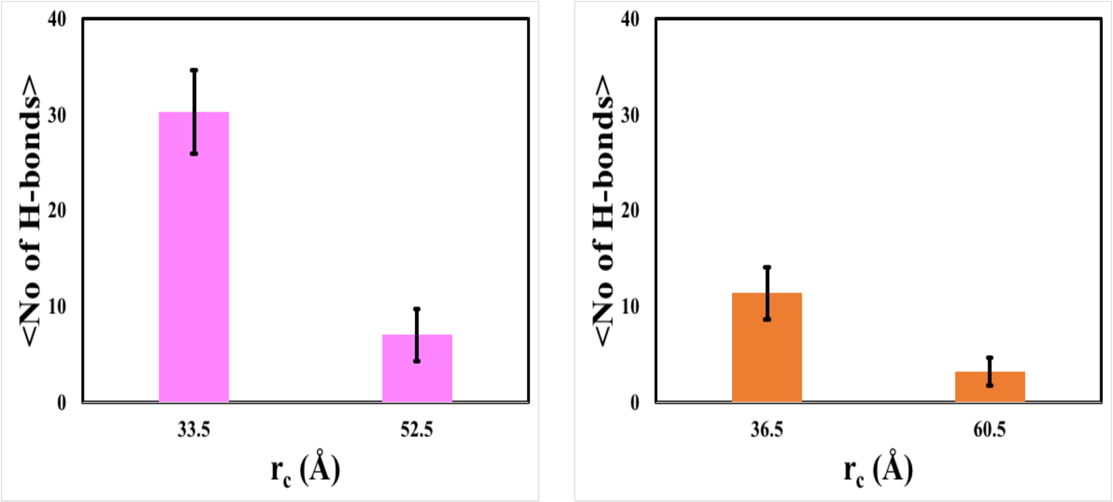

Figure S5. Average H-bonds when the **A.** nanofilament and the membrane **B.** nanotube and the membrane when nanotube/nanofilament is approaching the membrane and in contact with the membrane calculated for the last 10ns of the 28ns simulation.

References

1. Fiorin, G.; Klein, M. L.; Henin, J., Using Collective Variables to Drive Molecular Dynamics Simulations. *Molecular Physics* **2013,** *111* (22-23), 3345-3362.
2. Phillips, J. C.; Braun, R.; Wang, W.; Gumbart, J.; Tajkhorshid, E.; Villa, E.; Chipot, C.; Skeel, R. D.; Kale, L.; Schulten, K., Scalable Molecular Dynamics with Namd. *Journal of Computational Chemistry* **2005,** *26* (16), 1781-1802.
